# Measuring state-level infant and toddler well-being in the United States: Gaps in data lead to gaps in understanding

**DOI:** 10.1007/s12187-021-09902-4

**Published:** 2022-03

**Authors:** Renee Ryberg, Lisa Wiggins, Kristin A. Moore, Sarah Daily, Gabriel Piña, Ami Klin

**Affiliations:** 1 Child Trends, Chapel Hill, NC, USA; 2 Centers for Disease Control and Prevention, Atlanta, GA, USA; 3 Child Trends, Bethesda, MD, USA; 4 Marcus Autism Center, Emory University School of Medicine and Children’s Healthcare of Atlanta, Atlanta, GA, USA

**Keywords:** Infant/toddler, Early childhood well-being, Indicators

## Abstract

Children who are nurtured, protected, and supported in the first years of life tend to have better individual outcomes and are more likely to grow to become healthy, productive adults. Child well-being varies across states, yet the field lacks a comprehensive review of infant and toddler indicators measured at the state-level. This paper reviews indicators of well-being from the prenatal period to three years that meet certain a priori criteria. Most of the child-level indicators identified were in the physical health domain; relatively fewer indicators were found in the early cognition and language or social-emotional-behavioral domains. While some states are making progress toward developing integrated early childhood data systems, more work is needed to provide robust data on infant and toddler development. These results highlight the need to develop a broader range of indicators of infant and toddler well-being and improve measurement sources to better inform policies and programs advancing population health.

Equitable, prosperous, and sustainable societies are born from healthy children (Shonkoff & Phillips, 2000). Children who are nurtured, protected, and supported in the first years of life tend to have better individual outcomes and are more likely to grow to become more productive adults (Bailey et al., 2020; Center on the Developing Child, 2007; Center on the Developing Child, 2010; Shonkoff and Phillips, 2000). Yet not all children have an equitable start in life. Racial and ethnic disparities in well-being emerge even before birth (Wilkinson et al., 2021), and inequities by additional sociodemographic factors (e.g., family income, home language, and maternal education) across domains of development become evident at nine months and grow larger as infants become toddlers (Halle et al., 2009). These early years are crucial to healthy development due to the rapid changes in brain connectivity and skill acquisition that occur during this time (Masten & Cicchetti, 2010; Shonkoff & Phillips, 2000). Accordingly, the first three years have become a central focus of policies and programs designed to improve overall child well-being (Prenatal-to-3 Policy Impact Center, 2021).

Child well-being is globally defined as “the multi-dimensional nature of health that is enhanced when physical, cognitive, and social-emotional-spiritual development is nurtured in developmentally appropriate ways” (The Alliance for Child Protection in Humanitarian Action, 2021, p. 11). The well-being of children from the prenatal period to three years, henceforth referred to as “infant and toddler well-being,” varies across states (Keating et al., 2020). For instance, some states have higher infant mortality rates than others (CDC, 2021), which may lead to variations in policy and practice interventions. It is therefore important to develop indicators available for all states and the District of Columbia (DC) to help monitor how well communities fare compared to others on an outcome of interest at a single point in time (Szilagyi & Schor, 1998; Moore & Brown, 2003). Additionally, state-level indicators can be used to monitor trends in infant and toddler outcomes and enable researchers to assess associations between individual factors, policies and programs, and overall health and development.

Although associations with indicators cannot establish causality, they can inform whether policy interventions, such as expanding economic supports for disadvantaged families, are associated with better outcomes. Building on this understanding, indicators can help states set goals or targets for policies and programs, invest in advantageous policies and programs, and respond to the needs of families, educators, policymakers, and public health officials. Moreover, indicators can help states identify sub-populations of children who may be at risk for adverse outcomes, such as children and families who have faced racial discrimination and children of families with lower income (Keating et al., 2020; Wilkinson et al., 2021).

Efforts to identify indicators of child well-being have been undertaken in the United States since the 1970s (Lippman, 2007; Moore, 2020). Most indicator reports have tended to focus on a small set of negatively oriented indicators for preschool and school-aged children measured by well-established data collection systems (Moore, 2020; Moore et al., 2004). A major gap in the literature is a comprehensive review of both positive and negative indicators of infant and toddler well-being available for every state and DC that can inform policies and programs. The goal of this paper is, therefore, to review indicators of infant and toddler well-being that are publicly available, measured across states, representative of state populations, and measured over time. This review builds upon extant work by providing a conceptual framework, identifying a priori inclusion criteria for indicators and measurement sources, identifying gaps in measurement, and informing future efforts related to childhood indicator research.

## 1 Theoretical framework

**Fig. 1:**
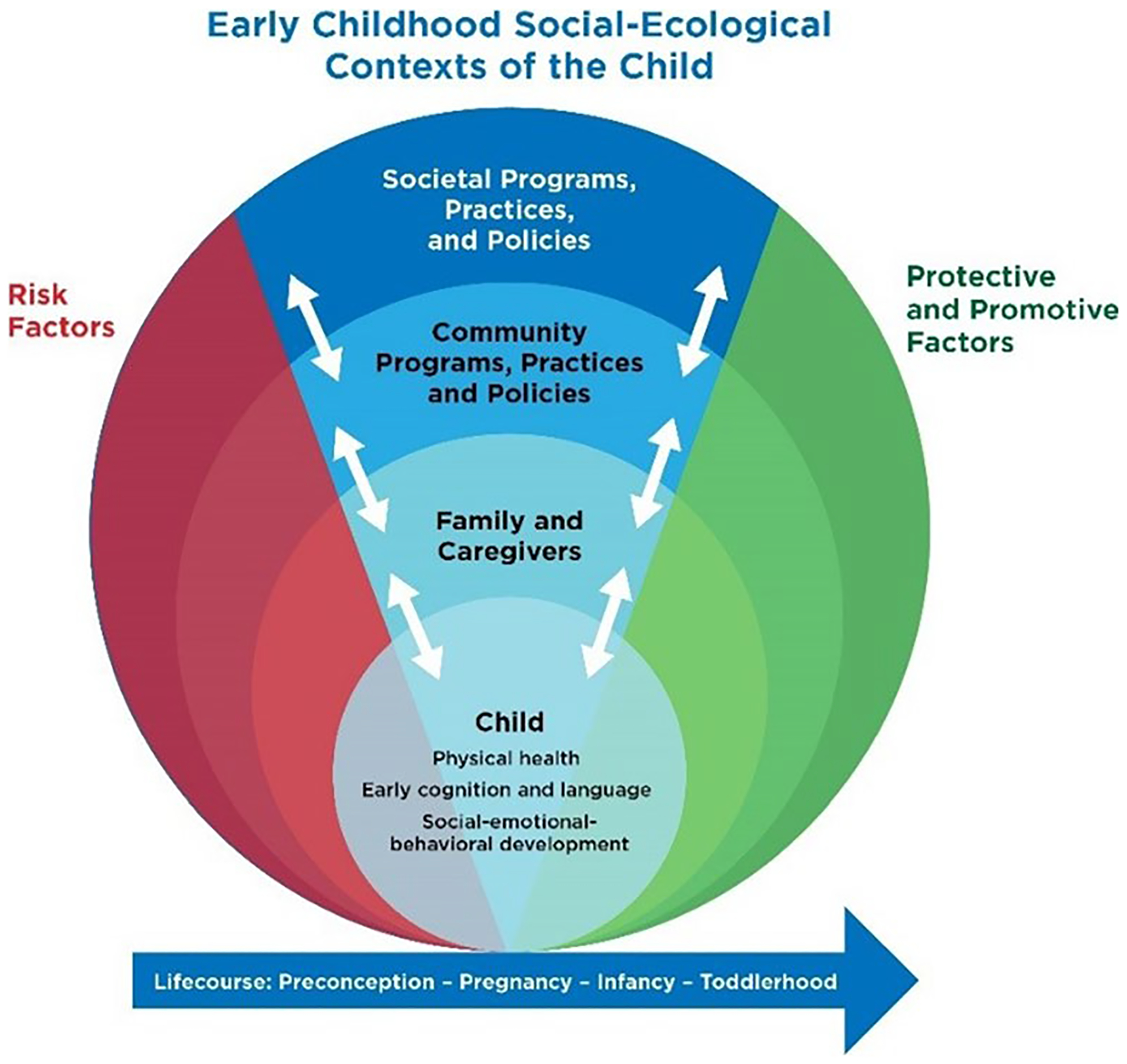
Framework for the identification of infant and toddler well-being indicators Note: This model was adapted from Bronfenbrenner (1979)’s social-ecological model for this paper.

Given an understanding that child development is a multi-faceted process continually influenced by internal and external forces that work together to shape the individual child, a social-ecological framework guides this review (Bronfenbrenner, 1979, 1992, 2007). The authors adapted the well-known social-ecological model to apply specifically to the infant and toddler context (see Fig. 1): Indicators at the individual level are influenced by indicators at the family, community, and societal levels. This paper focuses on indicators of infant and toddler well-being at the child level (the innermost circle) and the most proximal contextual factors that influence the child’s development and well-being (the family and caregivers circle). We did not focus on the neighborhood or the more distant contexts of development to limit the scope of the review to those most proximal to child development. We specifically focused on indicators of nurturing care identified by the World Health Organization, United Nations Children’s Fund, World Bank Group (2018): good health, adequate nutrition, responsive caregiving, security and safety, and opportunities for early learning. Within the span of child development, these indicators of infant and toddler well-being can be thought of as related to current child well-being. Infant and toddler well-being indicators are also predictive of well-being during later parts of the life course, or “well-becoming” as indicated by the arrow at the bottom of the figure (Ben-Arieh et al., 2001).

## 2 Methods

We took a four-step approach to review the state of measurement of infant and toddler well-being in the United States. We (1) developed criteria for identifying relevant indicators, (2) compiled a list of potential indicators using those criteria, (3) reviewed measurement sources for each potential indicator, and (4) solicited expert input on the list of indicators and their measurement sources. Each of these steps is described in more detail below.

### 2.1 Criteria for identifying potential indicators

Before identifying indicators of infant and toddler well-being, we first developed criteria for indicators to include in the review. Substantively, we were interested in measures of infant and toddler well-being that are relevant for children prenatally to age three, have a demonstrated connection to long-term outcomes, and focus on the child or their family or caregivers. We did not include more distal contextual indicators, such as those about the neighborhood or policy environments.

### 2.2 List of potential indicators

We next compiled a list of potential indicators of infant and toddler well-being that met the substantive criteria outlined above. Specifically, we conducted a focused review of academic literature in early child development as well as existing indicator tracking work from non-profit research organizations that have published data-driven advocacy or policy work, including the Zero To Three State of Babies Year-book (Keating et al., 2020), the University of Texas at Austin Prenatal-to-3 Policy Impact Center’s Roadmap (Prenatal-to-3 Policy Impact Center, 2020), the National Center for Children in Poverty’s Improving the Odds for Young Children State Early Childhood Profiles (National Center for Children in Poverty, 2018), and the Child Trends DataBank (Child Trends, 2020). The compiled list of all potential indicators based on this review is available in Appendix A.

### 2.3 Measurement sources for potential indicators

Then, we looked for sources of measurement for these potential indicators. Specifically, we looked for sources that are publicly available, comparable for all 50 states and DC, representative of state populations, and measured at regular time intervals (e.g., annually). The sources that met these criteria and were included in this review are outlined in Table 1.

### 2.4 Expert input on indicators and measurement sources

We convened two meetings of experts in August 2020 to solicit feedback on our process and preliminary findings. Experts were identified by the authors and invited to attend one of two meetings. The first group was comprised of experts on early childhood administrative data, and the second group was comprised of experts on early childhood development.^1^ Both groups provided input on relevant child and family/caregiver indicators as well as data quality and coverage. The experts agreed that our review had not missed any substantive indicators or major data sources, and their feedback is incorporated throughout this manuscript.

## 3 Results

Following the framework outlined in Fig. 1, we present the identified indicators of infant and toddler well-being that have measurement sources meeting our criteria categorized into three broad domains: physical health, early cognition and language, and social-emotional-behavioral development. Within each domain, we describe two types of indicators: those at the child-level and those at the family/caregiver level. Some of these indicators apply to multiple domains and have multiple measurement sources. See Appendices B–D for a complete listing of all indicators by developmental domain. This information is current as of Spring 2021.

### 3.1 Early physical health indicators

Many child health indicators are measured at birth, including birthweight (used to identify births with low birthweight); gestational age (used to identify preterm births); some health conditions of the newborn; some treatment received at birth (e.g., neonatal intensive care unit [NICU] admission, antibiotics); and infant mortality (Table 2). Data on these indicators are collected from birth and death certificates and are available through the CDC’s National Vital Statistics System (NVSS). From birth to 3 years, child indicators can be constructed using data on immunizations, physical health, oral health, sleep habits, and special health care needs using data from CDC’s National Immunization Survey (NIS) and the National Survey of Children’s Health (NSCH) (see Appendix B).

Family/caregiver indicators of infant and toddler *physical health* include whether the child is covered by health insurance (CPS), the adequacy and consistency of that insurance (NSCH), the food security of their household (CPS), whether the child received preventative care visits (NSCH), and whether the child has access to a medical home (NSCH). Information is also available on a series of indicators related to the mother’s health, including receipt of timely prenatal care and maternal health during pregnancy (NVSS) and data on breastfeeding initiation and duration (NIS). Finally, rates of child maltreatment, which may influence physical health other child outcomes, are available through the National Child Abuse and Neglect Data System (NCANDS).

### 3.2 Early cognition and language indicators

In the *early cognition and language* domain, data are available from the NSCH about diagnoses of developmental delays (Table 3). Data are also available from the NSCH for certain cognitive/language developmental milestones (e.g., is the child able to use two words together). Parents of children of all ages are asked about developmental delays whereas the latter set of items are asked about children who are at least 1 year old.

Family/caregiver indicators of infant and toddler well-being related to the *early cognition and language* domain include NSCH data on caregiver interactions with the child (e.g., reading to children singing and telling stories to children) and whether a health care provider asked about concerns related to the child’s learning or development.

### 3.3 Early social-emotional-behavioral indicators

The only nationally available child-level indicator of infant and toddler social-emotional-behavioral development is a battery of four items about flourishing designed to capture “curiosity and discovery about learning, resilience, attachment with parent(s), and contentment with life” (Table 4). These items are from the NSCH and asked of parents of children 0–5 years (Child and Adolescent Health Measurement Initiative, 2018, p. 55). Information on family/caregiver indicators that may influence social-emotional-behavioral development—such as exposure to adverse childhood experiences (ACEs), parental aggravation, maternal mental health, family resilience, and eating meals together—are also available through the NSCH. Additionally, Vital Statistics indicates whether a father is listed on birth certificates.

## 4 Discussion

In this paper, we sought to identify indicators of infant and toddler well-being from the prenatal period to three years that are publicly available, comparable for all 50 states and DC, representative of state populations, and measured at regular time intervals. Most of the child-level indicators we identified were in the *physical health* domain. Relatively fewer child-level indicators were found in the *early cognition and language* and *social-emotional-behavioral* domains. These results highlight the need to develop a broader range of indicators of infant and toddler well-being and improve measurement sources to better inform policies and programs advancing population health.

### 4.1 Strengths in existing infant and toddler indicators and measurement sources

The indicators of infant and toddler well-being included in this review focus primarily on infant and toddler *physical health*. Indicators of physical health are crucial, as poor infant health can have lifelong consequences (Black et al., 2017). For example, research from Canada has found that health at birth is associated with infant mortality in the short term and educational success and reliance on social programs in the long term (Oreopoulos et al., 2008).

NVSS is the federal compilation of birth and death certificates from the 50 states, five U.S. territories, New York City, and the District of Columbia. A wealth of information is collected from birth certificates, including birthweight and length of gestation, parental characteristics, and health of the newborn. The NVSS is the only source of national population-level birth data on newborns, and it is large enough to facilitate subgroup comparisons (e.g., by race/ethnicity) within states. However, the reliability and validity of birth certificate data vary widely by individual item and those on maternal tobacco and alcohol use, prenatal care, pregnancy complications, delivery events, and congenital malformations have been identified as items that need quality improvement (Northam & Knapp, 2006; Salemi et al., 2017). Additionally, analysts need to take into consideration changes in birth certificate designs over time when making historical comparisons.

The NIS and NSCH are surveys focused specifically on the health of children. The NIS-Child survey component is focused on immunization and breastfeeding histories among toddlers aged 19–35 months. The NSCH is more general—taking a whole child perspective—and captures health settings, behaviors, and outcomes for children ages 0–17 years. It is the primary source of information on the well-being of infants and toddlers beyond immunizations, breastfeeding, and birth experiences. Both the NSCH and NIS are annual data collections with data that can be compared across states. Together, they cover many well-being indicators relevant to infants and toddlers.

### 4.2 Challenges with existing infant and toddler indicators and measurements sources

Indicators of infant and toddler *early cognition and language* and *social-emotional-behavioral development* are more limited than those of *physical health*. Information needed to measure *early cognition and language* and *social-emotional-behavioral development* is frequently collected in clinical settings using child observations or parent questionnaires that capture rapid and interconnected changes in the early years. These clinical assessments are not easily scalable to population-level measurement due to the time and resource commitments needed for such data collection methods (Brito et al., 2019; Paschall et al., 2020). Nonetheless, NSCH has included questions on a child’s understanding and use of language and measures of flourishing for children 0–5 years. Ideally, additional measures of *early cognition and language* and *social-emotional-behavioral* skills appropriate to children from birth to three years will become more readily available in future years.

Many of the available indicators reflect the *absence* of positive development (e.g., infant mortality rates, reported cases of child maltreatment) rather than the *presence* of positive development (e.g., normative language development, demonstrating curiosity) or the *presence* of supportive contexts for young children’s development (e.g., positive parent-child or caregiver-child relationships). This dearth of “positive” indicators of child well-being has been an ongoing gap in the field for decades (Halle & Moore, 1998; Lippman, 2007; Lippman et al., 2014; Moore & Halle, 2001).

Even promising data sources have limitations. Sample sizes severely limit analysts’ ability to disaggregate data to examine disparities by race/ethnicity and income at the state level with the NIS and NSCH in a timely manner. For example, the CDC combines multiple years of NIS data to produce its state-level cohort estimates of breastfeeding rates, without looking at subgroups (Centers for Disease Control and Prevention, National Center for Chronic Disease Prevention and Health Promotion, Division of Nutrition, Physical Activity, and Obesity, 2021). Additionally, the public-use version of the NIS contains only four racial/ethnic group identifiers: Hispanic, non-Hispanic Black, non-Hispanic other, and non-Hispanic White, limiting analysts’ ability to examine smaller racial/ethnic subgroups (Centers for Disease Control and Prevention, National Center for Immunization and Respiratory Diseases, 2020). The last time the Current Population Survey produced estimates of food insecurity in households with children at the state level, it combined data from 2003 to 2011 to do so (Coleman-Jensen et al., 2013). With the NSCH, subgroup analyses are limited by sample size that is compounded by a survey redesign. The NSCH survey administration methodology was redesigned in 2016, and data before and after the redesign cannot be harmonized, limiting the number of years of data that can be combined (U.S. Census Bureau, 2021a).

Other data sources did not meet our inclusion criteria. For instance, family/caregiver indicators in the Behavioral Risk Factor Surveillance System (BRFSS) cannot be disaggregated for children from the prenatal period to age three. The Pregnancy Risk Assessment Monitoring System (PRAMS), National Health and Nutrition Examination Survey (NHANES), and National Health Interview Survey (NHIS) have indicators for infant and toddler well-being, but data are either not publicly available or not representative of state populations. Newborn screenings are not systemically measured so comparability across states is unclear. Further, administrative data can supply information on the number of children or families receiving varied services and benefits; however, these systems only include those receiving services and do not measure indicators for children who may be at-risk for adverse outcomes.

### 4.3 Ways to address challenges with child indicators and measurement sources

There are promising examples of ways to address the challenges with existing indicators and measurement sources highlighted in this report. In recent years, the World Health Organization developed the Global Scale for Early Development (GSED), a population-level measure of child development for children at birth to age three. The GSED is currently being validated in several countries, including the United States, with the goal of accelerating early childhood development monitoring (Black et al., 2019). Many European countries have population registries that provide basic data about all individuals, including infants and toddlers, that can be aggregated across communities. Data collected from these sources are shared with governments, schools, and community leaders to help make informed policy and program decisions that address the needs of all children.

Some states in the United States have also developed ways to address the challenges with existing child indicators and measurement sources. For example, several states have started a process to include home visiting data within early childhood integrated data systems (Lin, 2019). The long-term goal of these efforts is to gain a comprehensive understanding of child and family access to the programs and services infants and toddlers have received. Minnesota’s Early Childhood Longitudinal Data System (ECLDS) links data from the state departments of Education, Human Services, and Health. Using a public online platform, data users can access deidentified aggregate-level reports on topics such as birth records, program participation, financial assistance programs, and child and family demographics (Jordan et al., 2018). These efforts hold promise for linking administrative data with data about young children and their families, yet they are not at the stage where they include indicators of young children’s development.

Other communities across the United States are exploring ways to use developmental screeners such as the Ages and Stages Questionnaire (ASQ) to create a proxy for understanding whether infants and toddlers are developmentally “on track.” Developmental screeners cannot be used for diagnosis, but they can be effective in identifying the need for further assessment. Tracking assessment after developmental screening is complex and requires collaboration and coordination between practitioners, providers, and stakeholders. Efforts in Salt Lake City, Utah; Tarrant County, Texas; and Norwalk, Connecticut are being made to build partnerships, address data coordination challenges, and explore the viability of using screeners as population measures of infant and toddler development (K. Paschall, personal communication, November 4, 2021).

There may also be an opportunity to aggregate data in the United States in a similar fashion to European registries. Most infants and toddlers in the United States receive well-child check-ups (Keating et al., 2020). Information collected at these visits, such as timing of meeting developmental milestones, could theoretically be aggregated. This type of data linkage would be complex and would require integrating medical records across multiple data systems, using a shared data management platform, ensuring that data are de-identified in compliance with HIPAA, and following other relevant regulations. The de-identified, aggregate data source would then be a rich source of information on infant and toddler health and development.

### 4.4 Ways to prioritize future child indicator development and measurement efforts

We recognize that data collection efforts are extremely complex and expensive. There are ways to prioritize indicators collected through existing and upcoming data collection efforts when resources are limited. Here we focus on three considerations relevant to children’s outcomes: (i) prevalence, (ii) severity, and (iii) disparate impacts.

Children’s health has traditionally been assessed by evaluating indices that include the prevalence of *adverse* health conditions (e.g., proportion of newborns born small or too early), with a focus on discovering associations and finding effective prevention methods (NRC & IOM, 2004). The prevalence of typical child development is more prevalent than atypical child development, yet measures of appropriate developmental achievements appropriate for children from birth to three years are limited. Balancing indicators that measure adverse health outcomes, typical child development, and supportive contexts may help improve state-based monitoring for all children, including those who are at-risk versus presently demonstrating atypical development.

Another way of prioritizing the development of new indicators is to focus on factors that can severely impact subsequent development. Some measurement sources in this review collect data about conditions that can severely impact development, such as low birthweight or maltreatment. However, we lack comprehensive state-level measures on other potentially damaging conditions, such as malnutrition or psychosocial deprivation (Black et al., 2017). An important consideration when thinking about these types of indicators is whether social desirability bias prevents data from being accurately collected, as disclosing these conditions may be difficult for respondents.

Finally, some outcomes may be more impactful in vulnerable populations, raising equity concerns. A core principle of various indicators frameworks is to promote health equity (Raikes et al., 2017; Black et al., 2019). One example is the UNICEF “human rights-based approach” of selecting indicators for the child-related Sustainable Development Goals (SDG) that realize the rights of every child, especially the most disadvantaged. Measuring differences in well-being indicators across demographic subgroups can inform policy and program decisions to address health disparities. For instance, it is well known that maternal and child health inequities emerge even before birth in the United States (Wilkinson et al., 2021). Nationally representative studies find that measures of child development such as self-regulation or early learning skills vary by race and ethnicity by kindergarten (Piña et al., 2020), with differences even emerging by nine months (Halle et al., 2009). Yet, state-level data on these outcomes are limited for population subgroups.

Investing in resources to increase sample sizes and provide representative and comparable data across states could expanded opportunities for health disparity research. The NSCH has been able to increase its sample sizes through optional state oversamples, both of entire states and of more specific geographic areas. The NSCH costs approximately $16.82 per *sampled* address, but that is not the total cost per *response*, since an average of approximately six addresses must be sampled to achieve one completed response. Using a hypothetical example provided by the NSCH, it would cost a state about $58,870 to double their sample size (U.S. Census Bureau, 2021b). Among the 12 states that have or are currently sponsoring an oversample, actual costs for state oversamples have ranged from approximately $20,000 to $500,000 (A. Hirai, personal communication, October 22, 2021). Additionally, the CDC co-sponsored the 2021 NSCH in order to support a national increase in the sample size of children ages 1–5 years. The NSCH is also exploring options to improve state-level sample sizes for underrepresented racial/ethnic populations (A. Hirai, personal communication, October 22, 2021).

In other cases, current sampling procedures limit data sources’ ability to be easily expanded. The NIS, for example, is a phone-based survey targeting a very small proportion of the population (as respondents need to have a toddler ages 19–35 months for the child survey). The CPS Food Security Supplement is an add-on to the CPS and is dependent on the CPS sampling frame. Expanding the sample of either the NIS or the CPS Food Security Supplement would be very resource intensive. The NSCH is also exploring options to adapt its sampling to be able to target specific groups of children (A. Hirai, personal communication, October 22, 2021).

Recent national efforts have been established to prioritize equitable data collection and reporting. In January 2021, President Joe Biden signed an executive order to establish an Interagency Working Group on Equitable Data. This group will identify weaknesses in federal data collection efforts and help agencies expand and improve their data collection efforts (Exec. Order No. 13985, 2021). Additionally, the Robert Wood Johnson Foundation has established a National Commission to Transform Public Health Data Systems, aiming to rethink and improve the public health data system to promote health equity.

## 5 Conclusions

The first three years of life are critically important for a child’s development. State-level indicators can be used to monitor the well-being of infants and toddlers, monitor the equity of well-being across sub-populations, and inform policies and programming to promote child well-being. Indicators that are publicly available, comparable across states, representative of state populations, and measured at regular time intervals are primarily focused on physical health and lack indicators of infant and toddler development in other important domains essential to child well-being (early cognition and language and social-emotional-behavioral development; The Alliance for Child Protection in Humanitarian Action, 2021). While many states are making progress toward developing integrated early childhood data systems, more work is needed to provide robust data on indicators of nurturing care that facilitate infant and toddler development (World Health Organization, United Nations Children’s Fund, World Bank Group, 2018). Investing in comprehensive data collection efforts that balance adverse outcomes, typical child development, and supportive contexts may help improve state-based monitoring for all children. These data collection efforts could more fully support infants and toddlers as well as the parents, providers, and policymakers working to foster their development.

## Figures and Tables

**Table 1:** Infant and toddler measurement sources that have representative data for all 50 states and the District of Columbia and are measured at regular time intervals

Data source	Type	Description	Documentation reviewed^a^
American Community Survey (ACS)	Survey	Annual household-based survey conducted by the Census Bureau collecting basic demographic information	2019 ACS online codebook
National Vital Statistics System (NVSS; Vital Statistics)	Population	Compilation of data from birth and death certificates collected by the CDC	CDC Wonder^b^
Current Population Survey (CPS)	Survey	Monthly survey conducted by the Census Bureau and Bureau of Labor Statistics collecting information on labor force participation and select topics	2019 CPS online codebook
National Child Abuse and Neglect Data System (NCANDS)	Population	National database of child abuse and neglect compiled by the Administration for Children and Families	NCANDS child file codebook
National Immunization Survey-Child (NIS-Child)	Survey	Household survey conducted by the CDC focusing on children ages 19–35 months collecting information focused on immunization histories	NIS-Child codebook for the 2018 public-use data file
National Survey of Children’s Health (NSCH)	Survey	Annual household survey conducted by the Health Resources and Services Administration focused on children ages 0–17 collecting information on children’s health and home environments	2016–2017 NSCH codebook; 2017–2018 NSCH codebook; 2016, 2017, and 2018 NSCH questionnaires – Children Ages 0–5; Fast facts about the 2016–2017 NSCH; Fast facts about the 2017–2018 NSCH

a These are marked in the reference list with an *

b Vital statistics data were accessed through CDC Wonder. Additional data, including more historical data, may be available through the National Center for Health Statistics’ Vital Statistics Online Data Portal (https://www.cdc.gov/nchs/data_access/vitalstatsonline.htm)

**Table 2:** Select infant and toddler physical health indicators available at the child or family/caregiver levels

Indicator	Primary source
Child indicators	
*Low birthweight:* Percent of babies with birthweight less than 5.5 pounds	NVSS
*Preterm birth:* Percent of babies born before 37 completed weeks of gestation	NVSS
*Infant mortality rate:* Deaths under 1 year of age per 1000 live births	NVSS
*Abnormal conditions of newborn:* Percent of babies who experienced certain conditions at birth (assisted ventilation, NICU admission, surfactant replacement therapy, antibiotics for suspected neonatal sepsis seizures)	NVSS
*Congenital abnormalities:* Percent of babies who experienced congenital abnormalities (Anencephaly, Cleft Palate Alone, Cleft Lip with or without Cleft Palate, Cyanotic Congenital Heart Disease, Congenital Diaphragmatic Hernia, Omphalocele, Gastroschisis, Hypospadias, Meningocele/ Spina Bifida, Limb Reduction Defect, Down Syndrome, Suspected Chromosomal Disorder, Congenital Anomalies)	NVSS
*Immunizations:* Percent of toddlers ages 19–35 months who are up to date on recommended immunizations	NIS
*Special health care needs:* Percent of infants and toddlers who have special health care needs	NSCH
Family/caregiver indicators	
*Health insurance:* Percent of infants and toddlers with any health insurance	CPS
*Adequacy of health insurance:* Percent of infants and toddlers with health insurance that adequately covers their needs for the entire past 12 months	NSCH
*Prenatal care:* Percent of infants and toddlers whose mothers received timely prenatal care	NVSS
*Preventive medical visit:* Percent of infants and toddlers who saw a health care professional when they were not sick in the past 12 months	NSCH
*Medical home:* Percent of infants and toddlers who receive coordinated, ongoing comprehensive care within a medical home	NSCH
*Maternal health:* Percent of babies whose mothers experienced specific health conditions during pregnancy (eclampsia, gestational diabetes, gestational hypertension, infections)	NVSS
*Maternal mental health:* Percent of infants and toddlers whose mothers have less than optimal mental health	NSCH
*Breastfeeding initiation:* Percent of infants and toddlers who were ever breastfed	NIS
*Breastfeeding duration:* Percent of infants and toddlers who were breastfed more than 6 months	NIS
*Food security*: Percent of infants and toddlers living in food secure households	CPS Food Security Supplement
*Maltreatment:* Maltreatment rate per 1000 infants and toddlers	NCANDS

This is a select set of physical health indicators. See Appendix B for a full set of indicators and details about their measurement properties

**Table 3:** Select infant and toddler cognition and language indicators available at the child or family/caregiver levels

Indicator	Primary source
Child indicators	
*Cognitive/language development:* NSCH asks a series of items for children ages 1–5 about their ability to speak, form sentences, and ask questions, and follow directions.	NSCH
*Developmental Delays:* Percent of infants and toddlers whose parents have been told by a health care provider that they have a developmental delay	NSCH
Family/caregiver indicators	
*Developmental Surveillance:* Percent of infants and toddlers whose parents were asked by a health care provider about the child’s learning, development, or behavior	NSCH
*Developmental screening:* Percent of infants and toddlers whose parents filled out a developmental screener from a health care provider in the past year	NSCH
*Reading to children:* Percent of infants and toddlers who are read to every day	NSCH
*Singing and telling stories:* Percent of infants and toddlers who are told stories or sung songs to every day	NSCH

This is a select set of the most well-measured early cognition and learning indicators. See Appendix C for a full set of indicators and details about their measurement properties

**Table 4:** Select infant and toddler social-emotional-behavioral development indicators available at the child or family/caregiver levels

Indicator	Primary source
Child indicators	
*Flourishing:* Percent of infants and toddlers who meet the flourishing criteria based on a series of four items designed to capture curiosity and discovery about learning, resilience, attachment with parent, and contentment with life	NSCH
Family/caregiver indicators	
*Adverse childhood experiences:* Percent of infants and toddlers who have experienced at least one ACE (economic hardship, parent/guardian separated or divorced, parent/guardian died, parent/guardian incarcerated, witnessed abuse, witness or victim of neighborhood violence, lived with someone mentally ill, lived with someone with drug/alcohol abuse, treated with racial/ethnic bias)	NSCH
*Family resilience:* Percent of infants and toddlers who meet 4 family resilience items	NSCH
*Parental aggravation:* Percent of infants and toddlers whose parents seldom feel aggravation from parenting	NSCH
*Father on birth certificate:* Percent of babies for whom parents are married or there is paternal acknowledgement	NVSS

This is a select set of social-emotional-behavioral development indicators. See Appendix D for a full set of indicators and details about their measurement properties

## Footnotes

1 Experts included Leanne Barrett of Rhode Island Kid Count, Lindsey Bullinger of Georgia Tech, Tamara Halle of Child Trends, Erin Hardy of Data Diversity Kids and the Institute for Child Youth and Family Policy at Brandeis University, Amy Madigan at the Office of Planning, Research and Evaluation’s Division of Child and Family Development, David Murphey (independent consultant), William O’Hare (independent consultant), Michelle Osterman of the Centers for Disease Control and Prevention’s National Center for Health Statistics, Emily Putnam-Hornstein of the University of North Carolina at Chapel Hill, Holly Sexton of the University of Texas at Austin, and Jennifer Verbrugge of the Minnesota Department of Education.

## Appendix A. Potential indicators of infant and toddler development identified in literature review

**Table:**

Child indicators	Family/caregiver indicators^d^
*Physical health*	
Poor in-utero health	Maltreatment (abuse and neglect)
Low birthweight	Early childhood adversity^a^
Preterm birth	Exposure (victim/witness) to family-domestic violence (intimate partner violence, child maltreatment)
Abnormal conditions of newborn	Health insurance coverage
Congenital abnormalities	Medicaid coverage
APGAR score	Medical home
Infant mortality	Medical care visit
Overweight or underweight, obesity, weight-for-length	Gestational diabetes and maternal infections during pregnancy
Poor oral health	Insufficient folic acid during pregnancy
Physical health	Insufficient vitamin D during pregnancy
Physical activity	Pregnancy complications
Physical activity	Prenatal care
Immunizations	Maternal or paternal mental health
Chronic illness	Maternal smoking, drinking, or drug use
Unintentional injury	Maternal BMI (pre-natal)
Special health care needs (SHCN) status^b^	Maternal BMI (post-natal)
	Maternal mortality
	Maternal morbidity
	Breastfeeding initiation and duration
	Diet high in sugar and/or fat
	Malnutrition
	Food security
	Dental care visit
	Vision test
	Sleep conditions (consistency and position)
	Younger or older parental age
	Parental substance use
	Exposure to environmental toxins (e.g., pollutants in homes, second-hand smoke exposure, lead, organophosphates, mercury exposure/poisoning)
	Housing stability and quality^c^
	Unintended pregnancy
	Family planning
*Social-emotional-behavioral development*	
Flourishing (National Survey of Children’s Health [NSCH] definition: healthy attachment, curiosity, bouncing back, positive affect expression)	Adverse childhood experiences (ACEs): Caregiver exposure
Curiosity, exploration, or novelty-seeking	ACEs: Child exposure
Emotion regulation or expression skills	Exposure to child welfare system
Self-regulation skills	Shared family meals
Negative emotionality	Family resilience
Active temperament	Parental aggravation
Emotional withdrawal	Parental conflict
Approach, sociability, attention, or activity level	Family instability, turbulence
Attachment	Parenting (disengaged, punitive, harsh, authoritative; positive; co-parenting)
Reaching developmental milestones	Parental marital status
	Father on birth certificate
	Parental employment
	Parental education attainment
	Social capital
	Social support (family, friends)
	Unemployment rate
	Parent training in child development milestones
	Parent-child education program
*Early cognition and language*	
Cognitive/language development	Developmental surveillance
Developmental delay	Developmental screening
	Teacher training on children’s developmental stages
	Healthcare provider training on developmental screening
	Language-rich environment and caregiver communication (reading/singing/telling stories exposure)
	Excessive screen time (> 2 h per weekday)
	Access to high-quality, affordable child care
	High-quality ECE program dosage (days per week, months per year, etc.)
	Attendance in a high-quality early childhood education (ECE) program
	Child care instability
	Family poverty
	Family members with mental health issues or SHCN status

a Early childhood adversity is used as a catch-all term in the literature to capture all or some factors of adverse childhood experiences (ACES) and toxic stress. Early childhood adversities capture broad concepts (e.g., demographic, sociological, and prenatal exposures to trauma and developmental issues)

b We recognize the overlaps between developmental delay and special health care needs (SCHN) as well as the complexities of identifying and serving these needs for the 0–3 age group

c Housing stability and housing instability are catch-all terms that capture the impacts of issues such as crowding, frequency of moves, homelessness, and cost burden of home expenses

d Many of the child-level and family/caregiver indicators are related to multiple developmental domains. They are listed in the most relevant domain to save space

## Appendix B. Physical health indicators

**Table:**

Indicator	Sub-domain	Developmental stage	Data source	Definition by data source and notes	Years available and periodicity
Child indicators					
Low birthweight	Birth outcomes	Birth	NSCH	How much did he or she weigh when born? [72 oz or less to 155 oz or more] Notes: Major redesign in 2016, including new sampling method.	2016–present Annual
Low birthweight	Birth outcomes	Birth	NVSS birth certificate files^a^	Infant’s weight at birth is available in 3 sets of categories, through 8165 g, or Not stated: 12 groups in 500-g increments; 14 groups in 250-g increments; 100-g increments	1995–present Annual
Preterm birth	Birth outcomes	Birth	NSCH	Was this child born more than 3 weeks before his or her due date? [Yes; No] Notes: Major redesign in 2016, including new sampling method.	2016–present Annual
Preterm birth	Birth outcomes	Birth	NVSS birth certificate files^a^	Gestational age groups are available in 3 sets of categories, representing the duration of the pregnancy at the time of birth [in completed week increments]	1995–present Annual
Infant mortality rate	Mortality	Infant	NVSS death and linked birth/infant death certificate files^a^	The number of infant deaths (under 1 year old) per 1000 live births	1995–present Annual
Oral health	Oral health	Toddler	NSCH	How would you describe the condition of this child’s teeth? [This child does not have any teeth; Excellent; Very good; Good; Fair; Poor] Notes: Available for 1–3-year-olds. Major redesign in 2016, including new sampling method. May be part of combined school readiness measure	2016–present Annual
Oral health problems	Oral health	Toddler	NSCH	During the past 12 months, has this child had oral health problems such as toothaches, bleeding gums or decayed teeth or cavities, age 1–17 years?	2016–present Annual
				This measure was created based on three survey questions: 1. During the past 12 months, has (fill with CHILD’S NAME) had difficulty with or experienced any of the following? Decayed teeth or cavities [Yes; No] 2. During the past 12 months, has (fill with CHILD’S NAME) had difficulty with or experienced any of the following? Toothaches [Yes; No] 3. During the past 12 months, has this child had FREQUENT or CHRONIC difficulty with any of the following? Bleeding gums [Yes; No]	
				Notes: Available for 1–3-year-olds. Major redesign in 2016, including new sampling method	
Abnormal conditions of newborn	Physical health	Birth	NVSS birth certificate files^a^	Assisted ventilation, NICU admission, surfactant replacement therapy, antibiotics for suspected neonatal sepsis, seizures	1995–present Annual
APGAR scores	Physical health	Birth	NVSS birth certificate files^a^	APGAR scores are available for 5 min and 10 min after birth, in single increments or recoded into 5 categories: Single increments of 0–10; Unknown or Not Stated; Not Applicable. Recoded categories of A score of 0–3; A score of 4–6; A score of 7–8; A score of 9–10; Unknown or Not Stated.	1995–present
				Note that 10-min APGARS scores are categorized as “Not Applicable” when the 5-min score was 6 or higher, and reported as “Unknown or Not Stated” in the recoded categories.	
Congenital abnormalities	Physical health	Birth	NVSS birth certificate files^a^	Anencephaly, Cleft Palate Alone, Cleft Lip with or without Cleft Palate, Cyanotic Congenital Heart Disease, Congenital Diaphragmatic Hernia, Omphalocele, Gastroschisis, Hypospadias, Meningocele / Spina Bifida, Limb Reduction Defect, Down Syndrome, Suspected Chromosomal Disorder, Congenital Anomalies	1995–present Annual
Effect of conditions on daily activities	Physical health	Infant/toddler	NSCH	During the past 12 months, how often have this child’s health conditions or problems affected his or her ability to do things other children his or her age do? Mark ONE only. [This child does not have any conditions; Never; Sometimes; Usually; Always] Notes: Major redesign in 2016, including new sampling method. May be part of combined school readiness measure	2016–present Annual
Functional difficulties	Physical health	Infant/toddler	NSCH	This measure was scored as the count of difficulties children experience from a list of 12 functional difficulties During the past 12 months, whether the child had frequent or chronic difficulty with breathing or other respiratory problems; eating or swallowing; digesting food, including stomach/intestinal problems, constipation, or diarrhea; repeated or chronic physical pain, including headaches or other back or body pain; using his/her hands (0–5 years); coordination and moving around (0–5 years) Notes: Major redesign in 2016, including new sampling method. The 2018 measure did not change from 2017	2016–present Annual
Immunizations	Physical health	Toddler	NIS	Coverage of the following vaccinations: Diphtheria and tetanus toxoids and acellular pertussis vaccine (DTaP/DT/DTP); Polio-virus vaccine (Polio); Measles or Measles-Mumps-Rubella vaccine (MMR); Haemophilus influenzae type b vaccine (Hib); Hepatitis B vaccine (HepB); Varicella zoster (chickenpox) vaccine (VAR); Pneumococcal conjugate vaccine (PCV); Rota-virus vaccine (ROT); Hepatitis A vaccine (HepA); Influenza vaccine (Flu)	1995–present Annual
Injury/health issues	Physical health	Infant/toddler	NSCH	27 conditions were included in the scoring of this measure: allergies (food, drug, insect or other); arthritis; asthma; blood disorders (such as sickle cell disease, thalassemia, or hemophilia); brain injury/concussion/head injury; cerebral palsy; cystic fibrosis; diabetes; Down Syndrome; epilepsy or seizure disorder; genetic or inherited condition; heart condition; hearing problems; vision problems; frequent or severe headaches including migraine; Tourette Syndrome; anxiety problems; depression; behavioral and conduct problem; substance use disorder; developmental delay; intellectual disability; speech or other language disorder; learning disability (also known as mental retardation); other mental health condition; Autism or Autism Spectrum Disorder (ASD); Attention Deficit Disorder or Attention-Deficit/Hyperactivity Disorder (ADD or ADHD) Notes: Severity of conditions (list of 20) is also available Major redesign in 2016, including new sampling method. The information on whether the child still has a condition was modified in 2018	2016–present Annual
Physical Health: Overall health	Physical health	Infant/toddler	NSCH	In general, how would you describe this child’s health? [Excellent; Very Good; Good; Fair; Poor] Note: Major redesign in 2016, including new sampling method.	2016–present Annual
Physical Health: Ability to do things	Physical health	Infant/toddler	NSCH	To what extent do (fill with CHILD’S NAME)’s health conditions or problems affect his or her ability to do things? [Very little; Somewhat; A great deal] Notes: Major redesign in 2016, including new sampling method.	2016–present Annual
Special health care needs (SHCN) status	Physical health	Infant/toddler	NSCH	Does this child have special health care needs based on the CSHCN Screener? The NSCH uses the CSHCN Screener to identify children with special health care needs. To qualify as having special health care needs, the following criteria must be met: (a) The child currently experiences a specific consequence; (b) The consequence is due to a medical or other health condition; and (c) The duration or expected duration of the condition is 12 months or longer The first part of each screener question asks whether a child experiences one of five different health consequences: (1) use or need of prescription medication; (2) above average use or need of medical, mental health or educational services; (3) functional limitations compared with others of same age; (4) use or need of specialized therapies (occupational therapy, physical therapy, speech, etc.); and (5) treatment or counseling for emotional or developmental problems The second and third parts of each screener question ask those responding “yes” to the first part of the question whether the consequence is due to any kind of health condition and if so, whether that condition has lasted or is expected to last for at least 12 months All three parts of at least one screener question (or in the case of question 5, the two parts) must be answered “YES” in order for a child to meet CSHCN Screener criteria for having a special health care need Notes: Major redesign in 2016, including new sampling method. The 2018 item did not change from 2017	2016–present Annual
SHCN: Need prescribed medicine	Special health care needs	Infant/toddler	NSCH	Does this child CURRENTLY need or use medicine prescribed by a doctor, other than vitamins? [Yes; No] Notes: Major redesign in 2016, including new sampling method	2016–present Annual
SHCN: Services: health, mental, education	Special health care needs	Infant/toddler	NSCH	Does (fill with CHILD’S NAME) need or use more medical care, mental health, or educational services than is usual for most children of the same age? [Yes; No] Notes: Major redesign in 2016, including new sampling method	2016–present Annual
SHCN: Limited in ability	Special health care needs	Infant/toddler	NSCH	Is this child limited or prevented in any way in his or her ability to do the things most children of the same age can do? [Yes; No] Notes: Major redesign in 2016, including new sampling method	2016–present Annual
SHCN: Special therapy (physical, occupational, speech)	Special health care needs, physical health	Infant/toddler	NSCH	Does this child need or get special therapy, such as physical, occupational, or speech therapy? [Yes; No] Notes: Major redesign in 2016, including new sampling method.	2016–present Annual
SHCN: Treatment or counseling need	Special health care needs, mental health	Infant/toddler	NSCH	Does this child have any kind of emotional, developmental, or behavioral problem for which he or she needs treatment or counseling? [Yes; No] Notes: Major redesign in 2016, including new sampling method	2016–present Annual
Family/caregiver indicators^b^					
Health insurance	Health insurance	Infant/toddler	CPS	Any insurance (in past calendar year; at time of interview for some forms); Source of insurance [public or private; Any private insurance; Employer-sponsored insurance; Individually purchased insurance; Any public insurance; Any Medicaid/SCHIP/other public insurance; Medicare coverage; Any military insurance]	1962–present Annual
Health insurance	Health insurance	Infant/toddler	ACS	Any insurance at the time of interview; Source of insurance [private, employer, purchased directly, TRICARE, public, Medicaid, Medicare, VA, Indian Health Services]	2008–present Annual
Health insurance	Health insurance	Infant/toddler	NSCH	Is this child CURRENTLY covered by ANY kind of health insurance or health coverage plan? [Yes; No] Notes: Major redesign in 2016, including new sampling method	2016–present Annual
Adequate and continuous health insurance	Health insurance	Infant/toddler	NSCH	Is this child adequately and continuously insured; that is, is their current insurance adequate and were they insured for the entire past 12 months? [Children with adequate current insurance for their needs and insured entire year during the past 12 months; Children with current insurance not adequate for their needs and had a gap in insurance coverage during the past 12 months] Notes: Major redesign in 2016, including new sampling method	2016–present Annual
Adequacy of health insurance	Health insurance	Infant/toddler	NSCH	Is this child’s current insurance coverage usually/always adequate to meet his/her needs? [Child’s current insurance is adequate for child’s needs; Child’s current insurance is NOT adequate for child’s needs] Notes: Major redesign in 2016, including new sampling method	2016–present Annual
Consistency of health insurance coverage	Health insurance	Infant/toddler	NSCH	Did this child have consistent health insurance coverage during the past 12 months? [Children who have had continuous health insurance coverage during the past 12 months; Children who did not have health insurance or had periods of no coverage during the past 12 months] Notes: Major redesign in 2016, including new sampling method	2016–present Annual
Type of health insurance	Health insurance	Infant/toddler	NSCH	What type of health insurance coverage, if any, did the child have at the time of the survey? [Children with only public health insurance; Children with only private health insurance; Children with public and private health insurance; Children with no current insurance coverage] Notes: Major redesign in 2016, including new sampling method	2016–present Annual
Prenatal care	Health care experiences	Prenatal	NVSS birth certificate files^a^	Number of prenatal visits; Month maternal prenatal care began; Trimester prenatal care began	1995–present Annual
Forgone health care	Health care experiences	Infant/toddler	NSCH	During the past 12 months, was there any time when (fill with CHILD’S NAME) needed health care but it was not received? By health care, we mean medical care as well as other kinds of care like dental care, vision care, and mental health services. [Yes; No] Notes: Major redesign in 2016, including new sampling method	2016–present Annual
Medical care visit	Health care experiences	Infant/toddler	NSCH	2018: During the past 12 months, did this child see a doctor, nurse, or other health care professional for medical care (for example, preventive care, sick care, hospitalizations)? [Yes; No] 2016–2017: During the past 12 months, did (fill with CHILD’S NAME) see a doctor, nurse, or other health care professional for sick-child care, well-child check-ups, physical exams, hospitalizations or any other kind of medical care? [Yes; No] Notes: Major redesign in 2016, including new sampling method. Wording changed in 2018 so data are not comparable with previous years	2016–present Annal
Medical home (MH)	Health care experiences	Infant/toddler	NSCH	Children who receive coordinated, ongoing, comprehensive care within a medical home Notes: Major redesign in 2016, including new sampling method	2016–present Annal
MH: Usual source of care	Health care experiences	Infant/toddler	NSCH	Is there a place that this child USUALLY goes when he or she is sick or you or another caregiver needs advice about his or her health? [Yes; No] Where does this child USUALLY go first? Mark (X) ONE box. [Doctor’s Office; Hospital Emergency Room; Hospital Outpatient Department; Clinic or Health Center; Retail Store Clinic or “Minute Clinic;” School (Nurse’s Office, Athletic Trainer’s Office); Some other place] Notes: Major redesign in 2016, including new sampling method	2016–present Annual
MH: Personal doctor or nurse	Health care experiences	Infant/toddler	NSCH	Do you have one or more persons you think of as this child’s personal doctor or nurse? A personal doctor or nurse is a health professional who knows this child well and is familiar with this child’s health history. This can be a general doctor, a pediatrician, a specialist doctor, a nurse practitioner, or a physician’s assistant. [Yes; No] Notes: Major redesign in 2016, including new sampling method	2016–present Annual
MH: Referral for doctor or service	Health care experiences	Infant/toddler	NSCH	During the past 12 months, did this child need a referral to see any doctors or receive any services? [Yes; No] How much of a problem was it to get referrals? [Not difficult; Somewhat difficult; Very difficult; It was not possible to get a referral] Notes: Major redesign in 2016, including new sampling method	2016–present Annal
MH: Care coordination	Health care experiences	Infant/toddler	NSCH	Does anyone help you arrange or coordinate this child’s care among the different doctors or services that this child uses? [Yes; No] Overall, how satisfied are you with the communication among this CHILD’S doctors and other health care providers? [Very satisfied; Somewhat satisfied; Somewhat dissatisfied; Very dissatisfied] Overall, how satisfied are you with the health care provider’s communication with the school, child care provider, or special education program? [Very satisfied; Somewhat satisfied; Somewhat dissatisfied; Very dissatisfied] During the past 12 months, have you felt that you could have used extra help arranging or coordinating this CHILD’S care among the different health care providers or services? [Yes; No] During the past 12 months, how often did you get as much help as you wanted with arranging or coordinating this CHILD’S health care? [Yes; No] During the past 12 months, did this CHILD’S health care provider communicate with this CHILD’S school, child care provider, or special education program? [Yes; No] Notes: Major redesign in 2016, including new sampling method	2016–present Annual
MH: Family centered care	Health care experiences	Infant/toddler	NSCH	During the past 12 months, how often did this CHILD’S doctors or other health care providers: Spend enough time with this child? [Always; Usually; Sometimes; Never] During the past 12 months, how often did this CHILD’S doctors or other health care providers: Listen carefully to you? [Always; Usually; Sometimes; Never] During the past 12 months, how often did this CHILD’S doctors or other health care providers: Show sensitivity to your family’s values and customs? [Always; Usually; Sometimes; Never] During the past 12 months, how often did this CHILD’S doctors or other health care providers: Provide the specific information you needed concerning this child? [Always; Usually; Sometimes; Never] During the past 12 months, how often did this CHILD’S doctors or other health care providers: Help you feel like a partner in this CHILD’S care? [Always; Usually; Sometimes; Never] Notes: Major redesign in 2016, including new sampling method	2016–present Annual
Preventive medical visit	Health care experiences	Infant/toddler	NSCH	During the past 12 months, how many times did this child visit a doctor, nurse, or other health care professional to receive a PREVENTIVE check-up? A preventive check-up is when this child was not sick or injured, such as an annual or sports physical, or well-child visit. [0 visits; 1 visit; 2 or more visits] Notes: The lead-in question to this question changed between 2017 and 2018, precluding the comparison of results between the two years. Major redesign in 2016, including new sampling method	2016–present Annual
Maltreatment (abuse and neglect)	Maltreatment	Infant/toddler	NCANDS	Maltreatment rate per 1000 infants/toddlers	1995–present Annual
Maternal BMI (pre-natal)	Maternal health	Prenatal	NVSS birth certificate files^a^	7 categories for Body Mass Index: Underweight (<18.5); Normal (18.5–24.9); Overweight (25.0–29.9); Obesity I (30.0–34.9); Obesity II (35.0–39.9); Extreme Obesity III (>39.9); Unknown or Not Stated Notes: Maternal height, weight gain, and delivery weight are also available, so BMI at delivery could also be calculated	1995–present Annual
Pregnancy complications: Eclampsia	Maternal health	Prenatal	NVSS birth certificate files^a^	Eclampsia [Yes; No; Unknown or Not Stated; Not Reported]	1995–present Annual
Pregnancy complications: Gestational diabetes	Maternal health	Prenatal	NVSS birth certificate files^a^	Gestational Diabetes [Yes; No; Unknown or Not Stated]	1995–present Annual
Pregnancy complications: Gestational hypertension	Maternal health	Prenatal	NVSS birth certificate files^a^	Gestational Hypertension [Yes; No; Unknown or Not Stated]	1995–present Annual
Pregnancy complications: Maternal infections during pregnancy	Maternal health	Prenatal	NVSS birth certificate files^a^	Gonorrhea, Syphilis, Chlamydia, Hepatitis B, Hepatitis C, Infections Checked	1995–present Annual
Maternal morbidity	Maternal health	Birth	NVSS birth certificate files^a^	Maternal Transfusion, Third or Fourth Degree Perineal Laceration, Ruptured Uterus, Unplanned Hysterectomy, Admission to Intensive Care Unit, Maternal Morbidity Checked	1995–present Annual
Mothers reporting less than optimal mental health	Maternal health	Maternal	NSCH	Adult 1/2: In general, how is your mental or emotional health? [Excellent; Very Good; Good; Fair; Poor] Notes: Major redesign in 2016, including new sampling method.	2016–present Annual
Breastfeeding initiation	Nutrition	Infant	NVSS birth certificate files^a^	Infant breastfed at discharge	1995–present Annual
Breastfeeding initiation	Nutrition	Infant	NIS	Ever breastfed?	1994–present Annual
Breastfeeding initiation	Nutrition	Infant	NSCH	Was [CHILD’S NAME/this child ever breastfed or fed breast milk? [Yes; No] Notes: Major redesign in 2016, including new sampling method.	2016–present Annual
Breastfeeding duration	Nutrition	Infant	NIS	Duration of breastfeeding in days?	1994–present Annual
Breastfeeding duration	Nutrition	Infant	NSCH	How old was this child when he or she COMPLETELY stopped breastfeeding or being fed breast milk? [days, weeks, months] Notes: Major redesign in 2016, including new sampling method.	2016–present Annual
Food security	Nutrition	Infant/toddler	CPS Food Security Supplement (CPS-FSS)	Based on a series of 18 questions asked at the household level, households are categorized into four levels of food security based on USDA definitions: high food security, marginal food security, low food security, and very low food security. Note: Question wording changed in 1998. The supplement was also fielded from 1976 to 1977 and 1995–1997, but the data are not directly comparable.	1998–present Annual
Dental visit	Oral health	Toddler	NSCH	During the past 12 months, did this child see a dentist or other oral health care provider for any kind of dental or oral health care? [Yes, saw a dentist; Yes, saw other oral health care provider; No] Notes: Major redesign in 2016, including new sampling method.	2016–present Annual
Preventive dental care	Oral health	Toddler	NSCH	During the past 12 months, did this child see a dentist or other oral health care provider for preventive dental care, such as check-ups, dental cleanings, dental sealants, or fluoride treatments? [No preventive visits in past 12 months; Yes, 1 visit; Yes, 2 or more visits] Notes: Available for 1–3-year-olds. Major redesign in 2016, including new sampling method.	2016–present Annual
Vision test	Physical health	Infant/toddler	NSCH	2018: During the past 12 months, has this child had his or her vision tested with pictures, shapes, or letters? [Yes; No] 2016–2017: Has (fill with CHILD’S NAME) EVER had his or her vision tested with pictures, shapes, or letters? [Yes; No] Notes: Major redesign in 2016, including new sampling method.	2016–present Annual
Tobacco use in the house	Physical health	Infant/toddler	NSCH	Does anyone living in your household use cigarettes, cigars, or pipe tobacco? [Yes; No] Notes: Major redesign in 2016, including new sampling method.	2016–present Annual
Smoking inside the house	Physical health	Infant/toddler	NSCH	Does anyone smoke inside your home? [Yes; No] Notes: Major redesign in 2016, including new sampling method.	2016–present Annnual
Sleep consistency	Sleep	Infant/toddler	NSCH	How often does (fill with CHILD’S NAME) go to bed at about the same time on weeknights? [Always; Usually; Sometimes; Rarely; Never] Notes: Major redesign in 2016, including new sampling method.	2016–present Annual
Sleep position	Sleep	Infant	NSCH	In which position do you most often lay (fill with CHILD’S NAME) down to sleep now? Mark ONE only. [On his or her side; On his or her back; On his or her stomach] Notes: Available for 0–1-year-olds. Major redesign in 2016, including new sampling method.	2016–present Annual

a Vital statistics data were accessed through CDC Wonder (wonder.cdc.gov). Additional data, including more historical data, may be available through the National Center for Health Statistics’ Vital Statistics Online Data Portal (https://www.cdc.gov/nchs/data_access/vitalstatsonline.htm)

b Many of the family/caregiver indicators are related to multiple domains, but to streamline this manuscript, we placed the indicators in the most aligned domain

## Appendix C. Early cognition and language indicators

**Table:**

Indicator	Subdomain	Developmental stage	Data source	Definition by data source and notes	Years available and periodicity
Child indicators					
Cognitive/language development	N/A	Infant/toddler	NSCH	Is this child able to do the following…? Say at least one word, such as “hi” or “dog.” Use 2 words together, such as “car go.” Use 2 words together, such as “car go.” Use 2 words together, such as “car go.” Use 3 words together in a sentence, such as, “Mommy come now.” Ask questions like “who,” “what,” “when,” “where.” Ask questions like “why” and “how.” Tell a story with a beginning, middle, and end. Understand the meaning of the word “no.” Follow a verbal direction without hand gestures, such as “Wash your hands.” Point to things in a book when asked. Follow 2-step directions, such as “Get your shoes and put them in the basket.” Understand words such as “in,” “on,” and “under.” Notes: Major redesign in 2016, including new sampling method. These questions were asked to children ages 1–5 years only.	2016–present Annual
Developmental delays	N/A	Infant/toddler	NSCH	Has a doctor, other health care provider, or educator EVER told you that (fill with CHILD’S NAME) has…Developmental Delay? Examples of educators are teachers and school nurses. [Yes; No] Notes: Major redesign in 2016, including new sampling method.	2016–present Annual
Family/caregiver indicators^a^					
Developmental surveillance	N/A	Infant/toddler	NSCH	During the past 12 months, did this child’s doctors or other health care providers ask if you have concerns about this child’s learning, development, or behavior? [Yes; No] Notes: This is the often-forgotten half of the AAP guidance. Screening is not as effective without this surveillance. Major redesign in 2016, including new sampling method.	2016–present Annual
Developmental screening	N/A	Infant/toddler	NSCH	During the past 12 months, did a doctor or other health care provider have you or another caregiver fill out a questionnaire about observations or concerns you may have about this child’s development, communication, or social behaviors? Sometimes a child’s doctor or other health care provider will ask a parent to do this at home or during a child’s visit. [Yes; No] Notes: Available for ages 10 months – 3 years. Major redesign in 2016, including new sampling method.	2016–present Annual
Reading to children	N/A	Infant/toddler	NSCH	During the past week, how many days did you or other family members read to this child? [0 days; 1–3 days; 4–6 days; Every day] Notes: Major redesign in 2016, including new sampling method.	2016–present Annual
Singing and telling stories	N/A	Infant/toddler	NSCH	During the past 12 During the past week, how many days did you or other family members tell stories or sing songs to this child? [0 days; 1–3 days; 4–6 days; Every day] Notes: Major redesign in 2016, including new sampling method.	2016–present Annual
Screen time	N/A	Infant/toddler	NSCH	2018: ON MOST WEEKDAYS, about how much time did this child spend in front of a TV, computer, cellphone or other electronic device watching programs, playing games, accessing the internet, or using social media? [Less than 1 h; 1 h; 2 h; 3 h; 4 or more hours] 2016–2017: ON AN AVERAGE WEEKDAY, about how much time does (fill with CHILD’S NAME) usually spend in front of a TV watching TV programs, videos, or playing video games? [None; Less than 1 h; 1 h; 2 h; 3 h; 4 or more hours] 2016–2017: ON AN AVERAGE WEEKDAY, about how much time does (fill with CHILD’S NAME) usually spend with computers, cell phones, handheld video games, and other electronic devices, doing things other than schoolwork? [None; Less than 1 h; 1 h; 2 h; 3 h; 4 or more hours] Notes: Major redesign in 2016, including new sampling method.	2016–present Annual

a Many of the family/caregiver indicators are related to multiple domains, but to streamline this manuscript, we placed the indicators in the most aligned domain

## Appendix D. Social-emotional-behavioral development indicators

**Table:**

Indicator	Subdomain	Developmental stage	Data source	Definition by data source and notes	Years available and periodicity
Child indicators					
Flourishing	N/A	Infant/toddler	NSCH	For children age 0–5 years, four questions were asked that aimed to capture curiosity and discovery about learning, resilience, attachment with parent, and contentment with life. The survey question asked, “How true are each of the following statements about this child: (1) child is affectionate and tender, (2) child bounces back quickly when things don’t go his/her way, (3) child shows interest and curiosity in learning new things, and (4) child smiles and laughs a lot. The “Definitely true” response to the question indicates the child meets the flourishing item criteria. Notes: Available for 1–2-year-olds. The 2018 item cannot be combined with 2017 due to changes in items between the years. 2018 categories were: always, usually, sometimes, and never; whereas 2017 categories were: definitely true, somewhat true, and not true. Major redesign in 2016, including new sampling method.	2016–present Annual
Social-Emotional: Bouncing back	N/A	Infant/toddler	NSCH	2018: How often: Does this child bounce back quickly when things do not go his or her way? [Always; Usually; Sometimes; Never] 2016–2017: How well do each of the following phrases describe (fill with CHILD’S NAME)? (fill with CHILD’S NAME) bounces back quickly when things do not go his or her way? [Definitely true; Somewhat true; Not true] Notes: Question is the same from 2016 to 2018; however, the response options are different in 2018. Major redesign in 2016, including new sampling method.	2016–present Annual
Social-Emotional: Smile/laugh	N/A	Infant/toddler	NSCH	2018: How often: Does this child smile and laugh? [Always; Usually; Sometimes; Never] 2016–2017: How well do each of the following phrases describe (fill with CHILD’S NAME)? (fill with CHILD’S NAME) smiles and laughs a lot. [Definitely true; Somewhat true; Not true] Notes: Question is the same from 2016 to 2018; however, the response options are different in 2018. Major redesign in 2016, including new sampling method.	2016–present Annual
Social-Emotional: Affectionate/tender	N/A	Infant/toddler	NSCH	2018: How often: Is this child is affectionate and tender with you? [Always; Usually; Sometimes; Never] 2017–2016: How well do each of the following phrases describe (fill with CHILD’S NAME)? (fill with CHILD’S NAME) is affectionate and tender with you. [Definitely true; Somewhat true; Not true] Notes: Question is the same from 2016 to 2018; however, the response options are different in 2018. Major redesign in 2016, including new sampling method.	2016–present Annnual
Social-Emotional: Curiosity	N/a	Infant/toddler	NSCH	2018: Header: How often: Does this child show interest and curiosity in learning new things? [Always; Usually; Sometimes; Never] 2017–2016: Header: How well do each of the following phrases describe (fill with CHILD’S NAME)? (fill with CHILD’S NAME) shows interest and curiosity in learning new things. [Definitely true; Somewhat true; Not true] Notes: Question is the same from 2016 to 2018; however, the response options are different in 2018. Major redesign in 2016, including new sampling method.	2016–present Annual
Family/caregiver indicators^a^					
Adverse childhood experiences (ACEs): Child exposure	N/A	Infant/toddler	NSCH	Number of ACEs experienced by infants/toddlers. The ACEs included in the NSCH include: economic hardship, parent/guardian separated or divorced, parent/guardian died, parent/guardian incarcerated, witness of abuse, witness or victim of neighborhood violence, living with someone with mental health challenges, living with someone with alcohol/drug abuse, and being treated with bias due to race or ethnicity. Notes: Major redesign in 2016, including new sampling method.	2016–present Annual
ACEs: Economic hardship	N/A	Infant/toddler	NSCH	2018: SINCE THIS CHILD WAS BORN, how often has it been very hard to cover the basics, like food and housing, on your family’s income? [Never; Rarely; Somewhat often; Very often] 2016–17: SINCE THIS CHILD WAS BORN, how often has it been very hard to get by on your family’s income - hard to cover the basics like food or housing? [Never; Rarely; Somewhat often; Very often] Notes: Major redesign in 2016, including new sampling method. The wording of this individual item changed with 2018 and is not comparable across years; however, the overall ACE score can still be compared.	2016–present Annual
ACEs: Parent/guardian separated or divorced	N/A	Infant/toddler	NSCH	To the best of your knowledge, has this child EVER experienced any of the following? Parent or guardian divorced or separated [Yes; No] Notes: Major redesign in 2016, including new sampling method.	2016–present Annual
ACEs: Parent/guardian died	N/A	Infant/toddler	NSCH	To the best of your knowledge, has this child EVER experienced any of the following? Parent or guardian died [Yes; No] Notes: Major redesign in 2016, including new sampling method.	2016–present Annual
ACEs: Parent/guardian incarcerated	N/A	Infant/toddler	NSCH	To the best of your knowledge, has this child EVER experienced any of the following? Parent or guardian served time in jail [Yes; No] Notes: Major redesign in 2016, including new sampling method.	2016–present Annual
ACEs: Witnessed abuse	N/A	Infant/toddler	NSCH	To the best of your knowledge, has this child EVER experienced any of the following? Saw or heard parents or adults slap, hit, kick, punch one another in the home [Yes; No] Notes: Major redesign in 2016, including new sampling method.	2016–present Annual
ACEs: Witness or victim or neighborhood violence	N/A	Infant/toddler	NSCH	To the best of your knowledge, has this child EVER experienced any of the following? Was a victim of violence or witnessed violence in his or her neighborhood [Yes; No] Notes: Major redesign in 2016, including new sampling method.	2016–present Annual
ACEs: Lived with someone with mental health	N/A	Infant/toddler	NSCH	To the best of your knowledge, has this child EVER experienced any of the following? Lived with anyone who was mentally ill, suicidal, or severely depressed [Yes; No] Notes: Major redesign in 2016, including new sampling method.	2016–present Annual
ACEs: Lived with someone with alcohol/drug abuse	N/A	Infant/toddler	NSCH	To the best of your knowledge, has this child EVER experienced any of the following? Lived with anyone who had a problem with alcohol or drugs [Yes; No] Notes: Major redesign in 2016, including new sampling method.	2016–present Annual
ACEs: Treated with racial/ethnic bias	N/A	Infant/toddler	NSCH	To the best of your knowledge, has this child EVER experienced any of the following? Treated or judged unfairly because of his or her race or ethnic group [Yes; No] Notes: Major redesign in 2016, including new sampling method.	2016–present Annual
Shared family meals	N/A	Infant/toddler	NSCH	During the past week, on how many days did all the family members who live in the household eat a meal together? [0 days; 1–3 days; 4–6 days; Every day] Notes: Major redesign in 2016, including new sampling method.	2016–present Annual
Family resilience	N/A	Infant/toddler	NSCH	When your family faces problems, how often are you likely to do each of the following? (a) talk together about what to do, (b) work together to solve our problems, (c) know we have strengths to draw on, and (d) stay hopeful even in difficult times. [Children who live in a family that met 0–1 family resilience items; Children who live in a family that met 2–3 family resilience items; Children who live in a family that met all 4 family resilience items] Notes: Major redesign in 2016, including new sampling method	2016–present Annual
Parental aggravation	N/A	Infant/toddler	NSCH	Whether or not child lived with parents who often feel aggravation from parenting based on the following three items: During the past month, how often have you felt: That this child is much harder to care for than most children his or her age? [Never; Rarely; Sometimes; Usually; Always] That this child does things that really bother you a lot? [Never; Rarely; Sometimes; Usually; Always] Angry with this child? [Never; Rarely; Sometimes; Usually; Always] Notes: Major redesign in 2016, including new sampling method.	2016–present Annual
Father on birth certificate	N/A	Infant	NVSS birth certificate files^b^	Parents married or paternal acknowledgement Notes: Prior to 2016, CDC WONDER only has data on mother’s marital status. From 2016 to 2018, paternal acknowledgement is also included. Beginning in 2017, due to state statutory restrictions, California no longer provides record-level data on paternity acknowledgment for births occurring to California residents and non-residents (including residents of other states). For these births, Paternity Acknowledgment is reported as “Not Reported.”	2016–present Annual

a Many of the family/caregiver indicators are related to multiple domains. To streamline this manuscript, we placed the indicators in the most aligned domain

b Vital statistics data were accessed through CDC Wonder (wonder.cdc.gov). Additional data, including more historical data, may be available through the National Center for Health Statistics’ Vital Statistics Online Data Portal (https://www.cdc.gov/nchs/data_access/vitalstatsonline.htm)
